# Giant hepatic adenoma with bone marrow metaplasia not associated with oral contraceptive intake

**DOI:** 10.1186/1477-7819-4-58

**Published:** 2006-08-25

**Authors:** Giovanni Ramacciato, Giuseppe R Nigri, Paolo Aurello, Francesco D'Angelo, Francesca Pezzoli, Simone Rossi, Emanuela Pilozzi, Giorgio Ercolani, Matteo Ravaioli

**Affiliations:** 1 Hepatobiliary-pancreatic Surgery, Department of Surgery, University of Rome "La Sapienza", II School of Medicine, St. Andrea Hospital, Rome, Italy; 2 Department of Pathology, University of Rome "La Sapienza", II School of Medicine, St. Andrea Hospital, Rome, Italy; 3 Liver and Multivisceral Transplantation Unit, University of Bologna, St. Orsola-Malpighi Hospital, Bologna, Italy

## Abstract

**Background:**

Hepatocellular adenomas are the most common benign liver tumors. They are usually related to oral contraceptive intake.

**Case presentation:**

This case describes a 58-year-old woman admitted to our institution for a hepatic mass incidentally discovered during a routine examination. The patient, who was never on oral contraceptives, was asymptomatic upon admission. She underwent a thorough diagnostic evaluation and then a hepatic right trisegmentectomy. The histologic evaluation of the mass showed that it was a hepatocellular adenoma with areas of bone marrow metaplasia.

**Conclusion:**

Bone marrow metaplasia has rarely been found associated to liver tumors. The presence of marrow-derived hepatic progenitor cells might be the source of both adenoma hepatocytes and bone marrow differentiated cells. To our knowledge, this is only the second case in the English literature in which areas of bone marrow metaplasia were found in a hepatocellular adenoma.

## Background

Hepatocellular adenomas are considered the most common benign liver tumors. They are proliferative lesions arising from hepatocyte. They occur primarily in women between 20 to 40 years of age and are usually related to oral contraceptive intake. Even though the hepatic adenoma is not a malignant tumor, surgical intervention may be required to establish a histological diagnosis of the liver mass or if sudden massive bleeding or liver failure occurs. In the present case, the microscopic observation of the surgical specimen showed areas of bone marrow metaplasia within the lesion. To our knowledge, this is the second case in the English literature in which areas of bone marrow metaplasia were found in a hepatocellular adenoma. We, therefore, attempted to give an explanation to this observation.

## Case presentation

An asymptomatic 58 year-old woman was referred to this institution for a liver mass detected by abdominal ultrasonography (US) during a routine examination. The US showed a hypoechoic mass with calcifications and multiple non-homogeneous areas in the right hepatic lobe. Her past medical history was unremarkable. The menarche was at the age of 13, menstrual periods were regular. Physiologic menopause occurred when she was 49 years old. She had no history of drug or alcohol abuse. Physical examination was unremarkable.

Liver function tests and urine analysis were within normal limits. Hepatitis B surface antigen, anti-HBs antibody and anti-hepatitis C virus antibody were negative. Serum tumor markers (CEA, CA 19–9 and α-fetoprotein) were also negative. Moreover, laboratory and serology data ruled out liver abscess, amebae or hydatid cyst.

**Figure 1:**
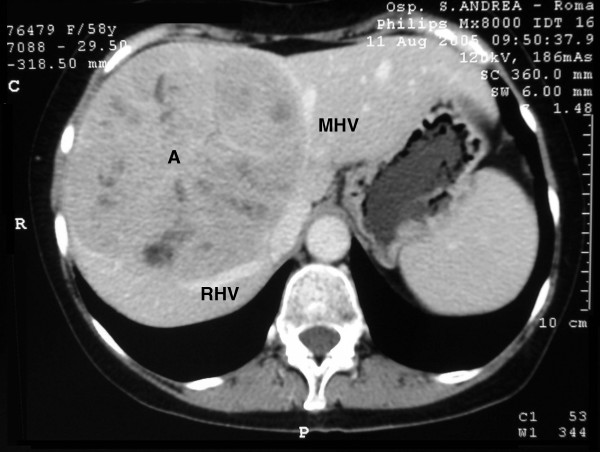
Contrast-enhanced CT scan. A: adenoma; Cy: cyst; C: calcifications within the cystic lesion (arterial phase);.

**Figure 2:**
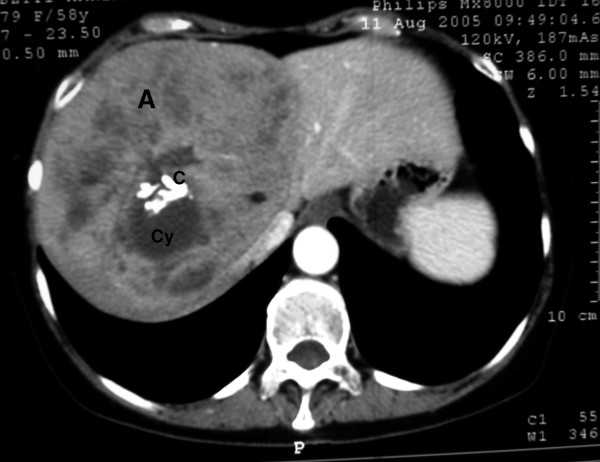
Contrast-enhanced CT scan. A: adenoma; RHV: right hepatic vein; MHV: middle hepatic vein (portal venous phase).

Abdominal computer tomography (CT) showed a voluminous lesion measuring 142 × 126 × 132 mm and localized in the IV, VII, and VIII hepatic segments Figure 1); it also showed the presence of multicystic areas with calcifications (Figure 2). The lesion was well defined, hypodense and with a hyperdense rim. The arterial phase exhibited significant enhancement during the arterial phase, decreasing during the portal phase, until becoming isodense relative to the liver in delayed scans. CT findings were characteristic for a giant hepatic adenoma. Since the diagnostic work-up supported the hypothesis of a hepatic adenoma, the surgical treatment was advocated. In this case surgery was the only therapeutic option. In fact, hepatic adenomas larger than 5 cm should be surgically removed due to the risk of hemorrhage and/or malignant transformation. No pre-operative biopsy was performed since its outcome would not have influenced the surgical treatment.

The patient was brought to the OR and a bilateral subcostal incision was performed. A voluminous hepatic lesion involving IV, VII and IVb segments was found. There was a first attempt to enucleate the mass from the surrounding liver parenchyma. However, since the mass was closely adherent to the right and middle hepatic veins, a liver resection was carried out. After performing the Pringle maneuver, parenchymal dissection was accomplished using the cavitational ultrasonic surgical aspirator. Hemostasis was achieved using argon beam coagulator, bipolar forceps and suture ligatures.

The specimen measured 19 × 15 × 7 cm. The mass appeared as a well circumscribed green-brown colored tumour with hemorrhagic areas and a cyst of 6 cm in diameter, with macroscopic calcification within. The surrounding liver tissue was normal.

**Figure 3:**
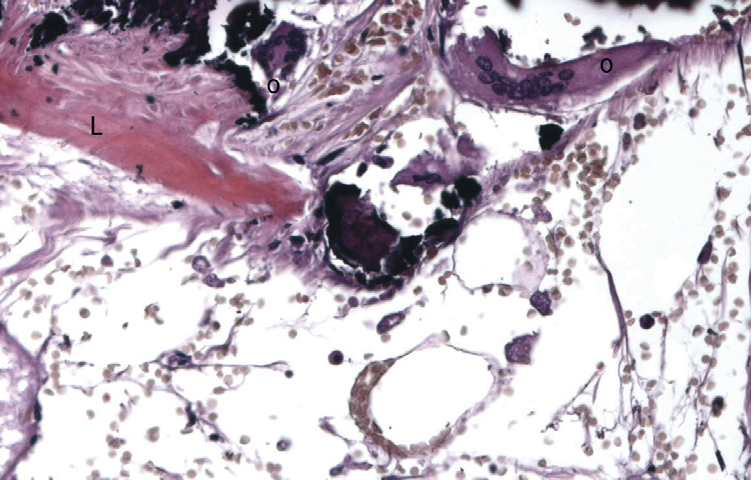
Optical microscopy (20×). Lamellar bone (L) found in the context of the fibrotic wall of a cyst present within the adenoma. Osteoclast (o – multinucleated cells) can be observed on the border of the trabeculae.

**Figure 4:**
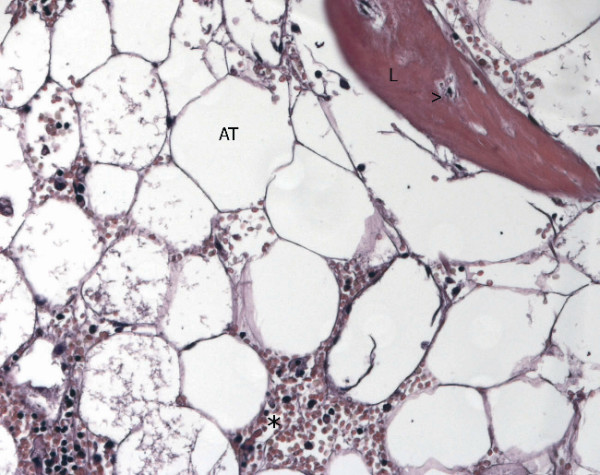
Optical microscopy (20×). Bone marrow metaplasia: lamellar bone forming a trabecula (L), adipose tissue (AT), and hemopoietic cells (*). Osteocytes are evident in the lacunae (>).

Microscopically, the lesion was composed of mature hepatocytes organized in sheets, mainly 2 cells thick. The cells had large cytoplasms and round nuclei with inconspicuous nucleoli. Some bi-nucleated cells were present. Gomori's staining showed that reticulin production was preserved within the proliferation. No vascular invasion was present. Large areas of hemorrhage and focal dilatation of peliosis-like sinusoid were present. A cystic formation with fibrotic and focally calcified wall was found within the lesion. Histologically, in the context of the wall, lamellar bone forming trabeculae intermingled with fat tissue containing myeloid and erythroid cells (bone marrow metaplasia) were found (Figure 3 and 4). CD 34 immunostaining was negative. Therefore, a diagnosis of hepatocellular adenoma was established.

The patient started oral intake on 4th postoperative day, with a carbohydrate-based diet. No signs of liver failure were recorded and the patient was discharged on day 13 post-op, after removing the J-P drains.

## Discussion

Among benign tumors of the liver, the hepatocellular adenoma is the most common. Its annual incidence is one case for 1 million persons per year. Three cases for 100,000 people per year occur among women who have had exposure to oral contraceptives. Risk increases with length of exposure to oral contraceptive[1,2]. Withdrawal of oral contraceptives is reported to induce the regression of the tumor, although this may take several months[3]. Another risk group for hepatic adenoma includes patients with glycogen storage disease: the prevalence is 50% in patients with type I glycogen storage disease and 25% with type III disease. In these patients, adenomas are also more likely to be multiple and to undergo malignant transformation [4]. Adenomas can also be found in association with other conditions such as diabetes mellitus, pregnancy, anabolic steroids, Fanconi anemia, Hurler disease, familial adenomatous polyposis and tyrosinemia.

Most of these tumors are detected incidentally by ultrasound or other scanning techniques. Others are discovered because of hepatomegaly, right upper quadrant discomfort, or intraperitoneal hemorrhage and the diagnosis is sometimes established only at laparotomy. Liver function tests are usually normal or only slightly elevated [5]. Ultrasound, CT scan and MRI can be employed as imaging techniques. In particular CT scan can show a well circumscribed and often encapsulated mass that has a low density on non-contrast phase, a marked centripetal pattern of enhancement on arterial phase and a central necrotic area or calcifications[6].

At gross examination, hepatic adenoma is typically a well circumscribed and encapsulated tumor with soft consistency and a size ranging from less than 1 cm to over 20 cm in diameter[7].

Microscopically, the hallmark of adenomas is the normal appearance of the hepatocytes. These are arranged in sheets and have no malignant features; these cells tend to be larger than normal hepatocytes and their cytoplasm often contain fat or glycogen. Areas of thrombosis and infarction may be observed.

In this case the unusual characteristic of the tumor was the presence of bone marrow metaplasia. The presence of bone marrow metaplasia could be explained by the effort of the liver to regenerate damaged hepatic tissue[8]. It has already been demonstrated that marrow-derived stem cells could be attracted by the damaged liver tissue upon releasing of cytokines and migration factors. At that point, stem cell could differentiate into hepatic progenitor cells and then into mature hepatocytes. Hepatic progenitor cells have been found in hepatic adenomas, as well as in hepatocellular carcinoma, hepatitis B virus induced cirrhosis, focal nodular hyperplasia, in small cell dysplastic foci and dysplastic nodules[9]. These observations indicate that hepatic progenitor cells may play a role in human liver tumor development. In conclusions, in our case it is possible to speculate that the presence of marrow-derived hepatic progenitor cells might be the source of both adenoma hepatocytes and bone marrow differentiated cells.

To our knowledge, this is the second case in the literature that bone marrow metaplasia has been associated with a hepatocellular adenoma. The first time it was discovered in a glycogen-storage disease-associated hepatic adenoma[10].

Differentiation of hepatocellular adenoma from high-grade hepatocellular carcinoma (HCC) can be difficult, and sometimes impossible. Adenoma tends to lack malignant-appearing mitotic structures and no cellular invasion into the capsule or surrounding liver parenchyma occurs. Unfortunately, these features may be absent in HCC, especially if it is well differentiated[7]. The diagnosis of hepatic adenoma is usually made when findings of malignancy are absent.

Even though the hepatic adenoma is not a malignant tumor, surgical intervention may be required to establish a histological diagnosis of the liver mass or if sudden massive bleeding or liver failure occur[11,12]. Because liver surgery had historically been associated with high rates of morbidity and mortality, non-operative management of benign liver lesions was commonplace. However, advances in anesthesia, surgical technique and post-operative care have produced dramatically improved outcomes after hepatic resection, allowing the routine inclusion of surgery as a therapeutic option[11,12]. Controversy remains only for hepatic adenomas smaller than 5 cm in diameter. In the present case surgical treatment was the only therapeutic option, considering that this voluminous adenoma could easily lead to massive hemorrhage or malignant transformation.

## Conclusion

It has been demonstrated that marrow-derived stem cells could be attracted by the damaged liver tissue upon releasing of cytokines and migration factors In conclusions, in our case it is possible to speculate that the presence of marrow-derived hepatic progenitor cells might be the source of both adenoma hepatocytes and bone marrow differentiated cells.

## Conflict of interest

The author(s) declare that they have no competing interests.

## Authors' contributions

**GR **designed the study and participated in the writing process

**GN **designed the study, carried out the data and picture acquisition as well as bibliographic research, drafted and revised the manuscript.

**PA, FA **participated in manuscript revision process.

**EP **performed histological assessment of the lesion and observed the presence of bone marrow metaplasia, provided the photomicrographs

**FP, SR, GE, and MR **they participated in the editing process.

All authors read and approved the final manuscript.
